# Association between glucagon and stroke in patients with type 2 diabetes

**DOI:** 10.1530/EC-25-0791

**Published:** 2026-02-17

**Authors:** Linlin Kong, Lina Chang, Jiamin Nie, Ying Liu, Yian Gu, Xin Wang, Siyu Yan, Wantong Han, Ming Liu, Qing He

**Affiliations:** Department of Endocrinology and Metabolism, Tianjin Medical University General Hospital, Tianjin, China

**Keywords:** glucagon, type 2 diabetes mellitus, stroke, risk indicator

## Abstract

**Objective:**

This study aimed to investigate the association between fasting glucagon levels and the risk of comorbid stroke in hospitalized patients with type 2 diabetes mellitus (T2DM).

**Methods:**

This study included 1,745 T2DM patients hospitalized at Tianjin Medical University General Hospital from September 1, 2022, to September 30, 2025. Patients were divided into a T2DM group and a T2DM with stroke group based on the presence of stroke. Fasting glucagon levels and other clinical data were collected. Binary logistic regression models were used to analyze the relationship between fasting glucagon and stroke risk.

**Results:**

Among female T2DM patients, fasting glucagon levels were significantly higher in the T2DM with stroke group compared to the T2DM group (13.38 vs 11.56 pmol/L, *P* = 0.011). Multivariable logistic regression analysis showed that after adjusting for multiple confounding factors, including age, diabetes duration, BMI, hypertension, eGFR, HbA1c, dyslipidemia, and medication use, higher fasting glucagon levels are independently associated with the presence of comorbid stroke in female T2DM patients (model 3: Q4 vs Q1: OR = 2.396, 95% CI: 1.075–5.339, *P* = 0.037). In addition, the prevalence of stroke increased with ascending quartiles of glucagon levels in female patients (*P* = 0.023). However, no significant association was observed between fasting glucagon levels and stroke risk in male patients.

**Conclusion:**

This study demonstrates that among hospitalized female patients with T2DM, higher fasting glucagon levels are independently associated with the presence of comorbid stroke. This association suggests a potential link between glucagon and cerebrovascular diseases in this population, warranting further investigation to explore its role.

## Introduction

Type 2 diabetes (T2DM), as a global health challenge, has become a focal point in international public health due to its continuously rising prevalence (1). Estimates from the International Diabetes Federation (IDF) indicate that in 2021, 537 million people worldwide had diabetes, leading to global healthcare expenditures of 966 billion US dollars, which are projected to exceed 1,054 billion US dollars by 2045 (2). Stroke, as one of the common macrovascular complications associated with T2DM, caused 6.55 million deaths in 2019, making it the second leading cause of death globally (3). The risk of ischemic stroke is increased by 2.5 times and the risk of hemorrhagic stroke by 1.5 times in patients with T2DM (4). This suggests that unique pathophysiological mechanisms exist between T2DM and cerebrovascular diseases, necessitating in-depth exploration.

The occurrence of stroke in T2DM patients results from the combined effects of multiple factors, such as age, gender, hypertension, smoking, and dyslipidemia (5, 6). However, beyond these traditional risk factors, endocrine hormones are also associated with the pathogenesis of macrovascular complications in type 2 diabetes (7). Glucagon, the primary counter-regulatory hormone to insulin, has long been considered mainly involved in the regulation of glucose and lipid metabolism and is in a state of hypersecretion in T2DM (8). However, given the widespread distribution of glucagon receptors in organs such as the liver, pancreas, kidneys, heart, and brain (9), its pathological role may be more extensive. Particularly, it is noteworthy that previous studies have indicated that increased glucagon levels are associated with an increased risk of diabetic kidney disease (10), prompting us to consider whether glucagon is similarly involved in the pathogenesis of macrovascular complications, such as stroke, in T2DM patients. However, clinical research evidence on the relationship between glucagon and type 2 diabetes complicated by stroke is currently lacking.

Considering this, this study, based on clinical data from inpatients with T2DM at Tianjin Medical University General Hospital from 2022 to 2025, investigates the association between fasting glucagon levels and the risk of type 2 diabetes complicated by stroke, aiming to provide new evidence for the early warning and targeted intervention of diabetic cerebrovascular complications.

## Methods

### Study subjects and research design

This study initially included a total of 2,450 type 2 diabetes patients hospitalized at Tianjin Medical University General Hospital from September 1, 2022, to September 30, 2025, who had their fasting glucagon levels measured. According to the exclusion criteria, 705 patients were excluded, resulting in 1,745 patients finally included in the study. Among the 305 patients with T2DM and stroke, 282 cases (92.5%) were ischemic stroke and 23 cases (7.5%) were hemorrhagic stroke. Diagnostic criteria were as follows: i) diabetes was defined as fasting plasma glucose ≥ 7.0 mmol/L, 2 h plasma glucose during an oral glucose tolerance test ≥ 11.1 mmol/L, random plasma glucose ≥ 11.1 mmol/L, glycated hemoglobin (HbA1c) ≥ 6.5%, self-reported history of diabetes, or use of glucose-lowering medication (11); ii) stroke diagnosis was based on the ‘Guidelines for the Early Management of Patients With Acute Ischemic Stroke’, utilizing patient clinical manifestations and imaging results, including head CT/MRI and head computed tomography angiography (CTA)/magnetic resonance angiography (MRA) (12); and iii) according to the ‘Chinese guidelines on prevention and treatment of dyslipidemia in adults 2016’, dyslipidemia was defined as total cholesterol (TC) ≥ 6.2 mmol/L, triglycerides (TG) ≥ 2.3 mmol/L, low-density lipoprotein cholesterol (LDL-C) ≥ 4.1 mmol/L, or high-density lipoprotein cholesterol (HDL-C) < 1.0 mmol/L, or being on lipid-lowering medication (13). Exclusion criteria were as follows: i) age < 18 years or pregnancy; ii) use of exogenous glucocorticoids; iii) severe hepatic or renal insufficiency, gastrointestinal diseases, anemia, and malignant tumors; iv) comorbidity with other endocrine diseases affecting blood glucose levels, such as hyperthyroidism; v) use of SGLT2 inhibitors; vi) acute stress events, such as acute stroke or myocardial infarction, occurring within the past 6 months; and vii) missing data.

### Collection of general information

Information from the electronic medical record system of T2DM inpatients was collected, including demographic characteristics, lifestyle, medical history, anthropometric measurements, laboratory test results, and medication information. Demographic characteristics included hospitalization number, gender, and date of birth; lifestyle information included smoking history and alcohol consumption history; medical history information included hypertension, coronary heart disease, hyperuricemia, fatty liver, and dyslipidemia; anthropometric measurements included height, weight, systolic blood pressure (SBP), and diastolic blood pressure (DBP); laboratory test results (test results were derived from peripheral venous blood collected in the fasting state on the morning following admission) included fasting plasma glucose, fasting C-peptide, fasting glucagon, TC, triglycerides (TG), high-density lipoprotein cholesterol (HDL-C), low-density lipoprotein cholesterol (LDL-C), glycated hemoglobin (HbA1c), and serum uric acid (SUA); and medication information included lipid-lowering drugs, antihypertensive drugs, GLP-1 receptor agonists, insulin, and dipeptidyl peptidase-4 inhibitors.

### Measurement of insulin, glucagon, and C-peptide

Plasma glucose was measured using the hexokinase method (instrument model: HITACHI 008AS, Japan). Insulin and C-peptide were measured using chemiluminescence immunoassay (instrument model: ARCHITECT i2000, USA). Pancreatic glucagon was measured using a chemiluminescence immunoassay method with dual monoclonal antibodies targeting the N and C termini of glucagon, employing a fully automated chemiluminescence analyzer (HomoG 100, China).

### Statistical methods and related formulas

Continuous variables conforming to a normal distribution are expressed as mean ± standard deviation, and Student’s *t*-test was used to compare differences between the two groups. Continuous variables not conforming to a normal distribution are expressed as median (interquartile range), and the Kruskal–Wallis rank sum test was used to compare differences between the two groups. Categorical variables are expressed as numbers (frequency), and the chi-square test was used to compare distribution differences between the groups. Multicollinearity among covariates was tested based on the variance inflation factor. Binary logistic regression was used for multivariate analysis, with stroke as the dependent variable, and covariates were progressively adjusted in three models. SPSS 27.0 (USA) was used for all statistical analyses; a two-tailed *P*-value <0.05 was considered statistically significant. The 2021 CKD-EPI eGFRcr equation was used (14).

## Results

### Clinical characteristics of T2DM and T2DM&Stroke patients

Table 1 presents the clinical characteristics of T2DM and T2DM complicated by stroke patients grouped according to gender. Supplementary Table 1 (see section on Supplementary materials given at the end of the article) shows the clinical characteristics of T2DM and T2DM complicated by stroke patients for the entire cohort. Compared to the T2DM group, the female T2DM&Stroke group was older (61 vs 68 years, *P* < 0.001), had a longer T2DM duration (96 vs 180 months, *P* < 0.001), had a higher prevalence of hypertension (54.9 vs 81.9%, *P* < 0.001), and also had higher frequencies of lipid-lowering drug and antihypertensive drug use (*P* all <0.001), leading to lower TC and LDL-C levels in the female T2DM&Stroke group (*P* < 0.001, *P* = 0.002, respectively).

**Table 1:** Clinical characteristics of patients with T2DM and T2DM&Stroke, grouped according to gender.

	Female			Male		
	T2DM	T2DM&Stroke	*P*	T2DM	T2DM&Stroke	*P*
Number, *n* (%)	567 (83.1)	116 (16.9)		873 (82.3)	189 (17.7)	
Age, years	61 (52, 69)	68 (63, 74)	***P* < 0.001**	54 (43, 64)	65 (60, 71)	***P* < 0.001**
T2DM duration, months	96 (12, 204)	180 (72, 252)	***P* < 0.001**	72 (13, 156)	132 (60, 240)	***P* < 0.001**
Smoking, *n* (%)	34 (6.0)	8 (6.9)	0.720	427 (48.9)	81 (42.9)	0.127
Alcohol consumption, *n* (%)	12 (2.1)	2 (1.7)	0.782	375 (43.0)	75 (39.7)	0.402
Hypertension, *n* (%)	311 (54.9)	95 (81.9)	***P* < 0.001**	488 (55.9)	149 (78.8)	***P* < 0.001**
Fatty liver, *n* (%)	393 (69.3)	78 (67.2)	0.679	630 (72.2)	184 (97.4)	***P* < 0.001**
Dyslipidemia, *n* (%)	404 (71.3)	90 (77.6)	0.229	714 (81.8)	163 (86.2)	0.241
Hyperuricemia, *n* (%)	113 (19.9)	29 (25.0)	0.262	264 (30.2)	46 (24.3)	0.088
Lipid-lowering drug, *n* (%)	180 (31.7)	60 (51.7)	***P* < 0.001**	287 (32.9)	105 (55.6)	***P* < 0.001**
Antihypertensive agents, *n* (%)	249 (43.9)	82 (70.7)	***P* < 0.001**	370 (42.4)	119 (63.0)	***P* < 0.001**
DPP-4i, *n* (%)	145 (25.6)	27 (23.3)	0.604	186 (21.3)	44 (23.3)	0.550
GLP-1RA, *n* (%)	53 (9.3)	15 (12.9)	0.240	128 (14.7)	24 (12.7)	0.481
Insulin, *n* (%)	308 (54.3)	65 (56.0)	0.736	414 (47.4)	109 (57.7)	**0.011**
BMI, kg/m^2^	25.39 (22.85, 28.58)	24.68 (23.09, 26.83)	0.099	26.73 (24.20, 29.70)	25.52 (23.54, 27.76)	***P* < 0.001**
BP, mmHg						
SBP	130 (121, 144)	132 (124, 146)	0.183	131 (120, 143)	136 (125, 148)	***P* < 0.001**
DBP	80 (74, 86)	78 (69, 86)	0.068	82 (76, 91)	82 (78, 90)	0.614
TC, mmol/L	4.97 (4.15, 5.84)	4.4 (3.66, 5.50)	***P* < 0.001**	4.77 (4.00, 5.56)	4.28 (3.34, 4.76)	***P* < 0.001**
TG, mmol/L	1.7 (1.22, 2.37)	1.69 (1.21, 2.42)	0.710	1.90 (1.34, 3.06)	1.43 (1.10, 2.12)	***P* < 0.001**
HDL-C, mmol/L	1.04 (0.89, 1.20)	1.01 (0.84, 1.19)	0.081	0.91 (0.80, 1.07)	0.91 (0.80, 1.10)	0.841
LDL-C, mmol/L	2.79 (2.20, 3.44)	2.46 (1.82, 3.20)	**0.002**	2.67 (2.07, 3.27)	2.25 (1.69, 2.81)	***P* < 0.001**
SUA, umol/L	299 (248, 369)	319 (253, 381)	0.248	354 (296, 434)	334 (277, 412)	**0.006**
eGFR, mL/min/1.73 m^2^	102.20 (93.89, 111.78)	94.53 (84.20, 101.24)	***P* < 0.001**	104.91 (95.33, 115.04)	95.94 (84.40, 103.30)	***P* < 0.001**
HbA1c, %	8.10 (7.10, 10.00)	7.85 (7.10, 9.33)	0.348	8.30 (6.90, 9.80)	8.00 (7.00, 9.00)	0.078

Bold indicates statistical significance (*P* < 0.05). Data are presented as mean ± SD, median (Q1, Q4), or number (%).

T2DM, type 2 diabetes mellitus; DPP-4i, dipeptidyl peptidase-4 inhibitors; GLP-1RA, glucagon-like peptide-1 receptor agonists; BMI, body mass index; BP, blood pressure; SBP, systolic blood pressure; DBP, diastolic blood pressure; TC, total cholesterol; TG, triglycerides; HDL-C, high-density lipoprotein cholesterol; LDL-C, low-density lipoprotein cholesterol; SUA, serum uric acid; and HbA1c, glycated hemoglobin.

The male T2DM&Stroke group was older (54 vs 65 years, *P* < 0.001), had a longer T2DM duration (72 vs 132 months, *P* < 0.001), had higher prevalence rates of hypertension and fatty liver (55.9 vs 78.8%, *P* < 0.001; 72.2 vs 97.4%, *P* < 0.001), and also had higher frequencies of lipid-lowering drug and antihypertensive drug use (*P* all <0.001). Furthermore, the male T2DM&Stroke group had a lower BMI, TC, TG, LDL-C, eGFR (*P* all <0.001), and SUA (*P* = 0.006).

### Analysis of differences in fasting glucagon, blood glucose, C-peptide, and insulin

For females, fasting glucagon levels (13.38 vs 11.56, *P* = 0.011) and C-peptide levels (2.35 vs 1.79, *P* = 0.035) were higher in the T2DM&Stroke group. For males, no significant differences were found in fasting glucagon levels between the T2DM and T2DM&Stroke groups. Fasting blood glucose levels (7.50 vs 6.85, *P* < 0.001) and C-peptide levels (1.90 vs 1.66, *P* = 0.042) were lower in the T2DM&Stroke group (Table 2).

**Table 2:** Analysis of differences in fasting glucagon, blood glucose, c-peptide, and insulin.

	T2DM	T2DM&Stroke	*P*
Female			
GCG0, pmol/L	11.56 (8.74, 16.25)	13.38 (9.35, 19.64)	**0.011**
FBG, mmol/L	7.5 (5.90, 10.00)	7.00 (6.15, 9.75)	0.520
FC-P, ng/mL	1.79 (1.10, 2.69)	2.35 (1.09, 3.31)	**0.035**
FINS, mU/L	12.50 (8.10, 21.20)	15.45 (8.20, 30.63)	0.131
Male			
GCG0, pmol/L	15.34 (10.94, 22.53)	14.18 (10.52, 20.15)	0.089
FBG, mmol/L	7.50 (6.10, 9.70)	6.85 (5.73, 8.40)	***P* < 0.001**
FC-P, ng/mL	1.90 (1.26, 3.00)	1.66 (0.92, 2.85)	**0.042**
FINS, mU/L	15.34 (10.94, 22.53)	11.95 (6.50, 22.13)	0.916

Bold indicates statistical significance (*P* < 0.05). Data are presented as median (Q1, Q4).

T2DM, type 2 diabetes mellitus; T2DM&Stroke, type 2 diabetes mellitus complicated by stroke; GCG0, fasting glucagon; FBG, fasting blood glucose; FC-P, fasting C-peptide; and FINS, fasting insulin.

**Figure 1:**
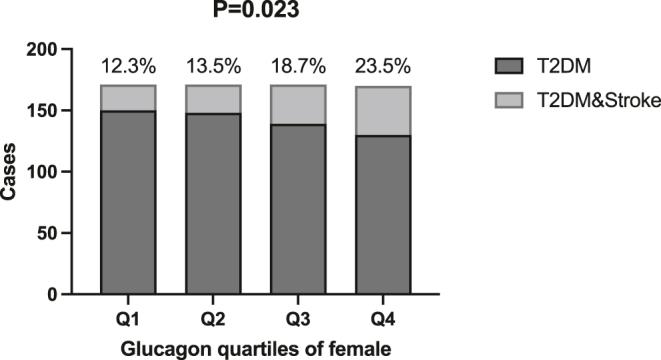
Distribution of female patients grouped by quartiles of fasting glucagon levels. Note: this figure shows the distribution of the number of female type 2 diabetes patients (*n* = 683) included in the study, grouped by quartiles (Q1–Q4) of their fasting glucagon (GCG) levels. Q1–Q4 represent groups from the lowest to the highest glucagon levels, respectively. The x-axis represents the percentiles of glucagon levels, and the y-axis represents the corresponding number of patients. The stroke prevalence rates for Q1, Q2, Q3, and Q4 groups were 12.3, 13.5, 18.7, and 23.5%, respectively. *P* < 0.05 was considered statistically significant.

**Figure 2:**
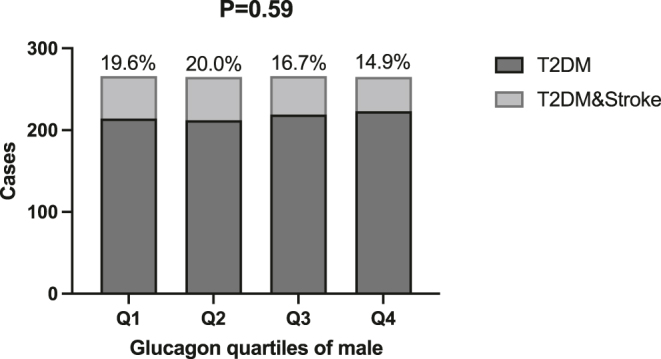
Distribution of male patients grouped by quartiles of fasting glucagon levels. Note: this figure shows the distribution of the number of female type 2 diabetes patients (*n* = 1,062) included in the study, grouped by quartiles (Q1–Q4) of their fasting glucagon (GCG) levels. Q1–Q4 represent groups from the lowest to the highest glucagon levels, respectively. The x-axis represents the percentiles of glucagon levels, and the y-axis represents the corresponding number of patients. The stroke prevalence rates for Q1–Q4 groups were 19.6, 20.0, 16.7, and 14.9%, respectively. *P* < 0.05 was considered statistically significant.

In female T2DM patients, the prevalence of stroke increased with increasing quartiles of glucagon (*P* = 0.023), whereas no statistically significant difference in stroke prevalence was observed in male patients. See Figs 1 and 2.

### Analysis of fasting glucagon in T2DM patients and T2DM&Stroke patients

Supplementary Table 2 provides the VIF values for each covariate. The relationship between fasting glucagon levels and the risk of T2DM complicated by stroke was examined separately for females and males using multivariate logistic regression models with progressive adjustment for influencing factors across three models. For female patients, model 1 was unadjusted; model 2 was adjusted for age and T2DM duration based on model 1; and model 3 was further adjusted for BMI, fatty liver, hypertension, eGFR, fasting insulin, HbA1c, dyslipidemia, use of antihypertensive agents, use of GLP-1 receptor agonists, use of DPP-4 inhibitors, and use of insulin based on model 2. After these adjustments, elevated plasma glucagon levels remained significantly associated with an increased risk of stroke (model 1: Q4 vs Q1: OR, 2.885, 95% CI, 1.396–5.961, *P* = 0.004; model 2: OR, 3.036, 95% CI, 1.431–6.443, *P* = 0.003; and model 3: OR, 2.396, 95% CI, 1.075–5.339, *P* = 0.037). No significant relationship was found between fasting glucagon levels and the risk of T2DM complicated by stroke in males. See Table 3.

**Table 3:** Binary logistic regression analysis of fasting glucagon in patients with T2DM and T2DM&Stroke.

	OR (95% CI)		
	Model 1	Model 2	Model 3
Female			
GCG, pmol/L	1.053 (1.020, 1.087)**	1.060 (1.024, 1.097)**	1.052 (1.014, 1.091)**
Per-SD increase	1.548 (1.181, 2.028)**	1.632 (1.218, 2.187)**	1.530 (1.121, 2.089)**
Q1	Reference	Reference	Reference
Q2	1.321 (0.596, 2.927)	1.225 (0.539, 2.786)	1.097 (0.463, 2.601)
Q3	1.474 (0.663, 3.274)	1.457 (0.639, 3.324)	1.139 (0.477, 2.722)
Q4	2.885 (1.396, 5.961)**	3.036 (1.431, 6.443)**	2.396 (1.075, 5.339)*
Male			
GCG, pmol/L	0.985 (0.963, 1.008)	0.995 (0.971, 1.019)	0.989 (0.963, 1.015)
Per-SD increase	0.880 (0.727, 1.066)	0.956 (0.781, 1.169)	0.911 (0.730, 1.136)
Q1	Reference	Reference	Reference
Q2	1.348 (0.787, 2.307)	1.439 (0.804, 2.576)	1.459 (0.794, 2.680)
Q3	1.086 (0.631, 1.868)	1.329 (0.742, 2.379)	1.310 (0.713, 2.406)
Q4	0.791 (0.440, 1.420)	1.011 (0.539, 1.897)	0.876 (0.448, 1.711)

Glucagon level quartiles of female: Q1 (<8.91), Q2 (8.91–11.71), Q3 (11.72–16.65), and Q4 (≧16.66).

Glucagon level quartiles of male: Q1 (<10.89), Q2 (10.89–15.05), Q3 (15.06–22.21), and Q4 (≧22.22).

Bold indicates statistical significance (*P* < 0.05). Significance levels:*P < 0.05, **P < 0.01.

Model 1: no adjustment was made.

Model 2: adjusted for age and T2DM disease duration based on model 1.

Model 3: adjusted for BMI, fatty liver, hypertension, eGFR, fasting insulin, HbA1c, dyslipidemia, antihypertensive agents, use of GLP-1RA, use of DPP-4i, and use of insulin based on model 2.

## Discussion

This study, based on a cross-sectional design, explored the association between fasting glucagon levels and the risk of comorbid stroke in hospitalized T2DM patients. Our main finding is that among female T2DM patients, higher fasting glucagon levels were significantly positively associated with the prevalence risk of stroke, and this association persisted after progressively adjusting for various confounding factors, including demographic characteristics, metabolic indicators, and medication history. However, we did not observe a similar significant association in male patients. This result suggests that glucagon may have a unique link with cerebrovascular complications in female T2DM patients.

The findings of this study provide new clinical evidence expanding the role of glucagon in macrovascular complications of T2DM. Traditionally, the role of glucagon in the pathophysiology of T2DM has been primarily defined as driving hepatic glucose output, leading to fasting hyperglycemia (15). However, the widespread distribution of glucagon receptors, particularly their expression in tissues such as the brain, heart, and blood vessels, suggests that its physiological functions extend far beyond the regulation of glucose and lipid metabolism. This view is supported by some cutting-edge research: for example, a Mendelian randomization study conducted by Ng *et al.* in the general population suggested that genetically determined higher glucagon levels might be a potential risk factor for ischemic heart disease (16). This provides genetic evidence for the cardiovascular effects of glucagon beyond traditional risk factors. On the other hand, within the field of diabetes, previous studies have begun to focus on the association between glucagon and microvascular complications; for example, some cross-sectional studies indicate that increased glucagon levels are independently associated with an increased risk of diabetic kidney disease (10, 17). Our study found an association between glucagon and the risk of comorbid stroke in the female T2DM population, which logically forms a beneficial extension: namely, the potential pathological role of glucagon may involve both microvascular and macrovascular complications. This study is the first to extend this association specifically to the cerebrovascular field at the clinical level, thereby supporting the hypothesis that glucagon may be broadly involved in the occurrence of vascular complications in T2DM.

This study found the presence of sex heterogeneity. Why does this association exist only in females and not in males? We speculate that there may be several reasons: first, at the socio-behavioral level, traditional gender roles may influence health behavior patterns. Men have higher exposure rates to lifestyle risk factors, including smoking and alcohol consumption (18, 19), and the effects of these factors might mask the relatively weaker effect of glucagon. Second, the sex hormone environment, particularly estrogen levels, may play a regulatory role. Estrogen exerts endothelial protective effects in the early stage of vascular injury by inhibiting vascular wall inflammatory responses (such as downregulating VCAM-1, ICAM-1, and other adhesion molecules, and the NF-κB signaling pathway), reducing leukocyte infiltration, and regulating the interaction between smooth muscle cells and adventitial fibroblasts in an ERα- and ERβ-dependent manner (20, 21). On the other hand, isolated islet experiments suggest that estrogen directly inhibits low glucose-induced glucagon secretion in pancreatic α-cells through a non-classical membrane estrogen receptor (22). In animal experiments, estrogen reduces plasma glucagon levels in rats, suggesting a direct inhibitory effect on pancreatic α-cells (23). The T2DM patients with stroke in this study were older, with a median age of 66 years, suggesting that most female patients were already in a postmenopausal state with significantly decreased estrogen levels. This may concurrently impair vascular protective mechanisms while relieving the inhibition of glucagon secretion, thereby making the association between hyperglucagonemia and vascular disease more pronounced. In males, estrogen levels are consistently low without a similar abrupt change process such as menopause; therefore, a significant correlation similar to that observed in females was not observed. Furthermore, previous research has shown that SGLT2 inhibitors increase fasting and postprandial glucagon levels, whereas GLP-1 receptor agonists and DPP-4 inhibitors can decrease fasting and postprandial glucagon levels. Meanwhile, the effect of exogenous insulin on glucagon varies greatly among individuals (24). Considering sample size issues, this study only excluded patients using SGLT2 inhibitors; perhaps, the use of GLP-1 receptor agonists and DPP-4 inhibitors contributed to a false negative for this association in male T2DM patients. Clarifying the intrinsic mechanisms of this sexual dimorphism requires further in-depth exploration in future studies.

From a clinical practice perspective, our findings suggest that for female T2DM patients, fasting glucagon levels may have the potential to serve as an auxiliary biomarker to help identify individuals at a higher risk of stroke. Assessing glucagon levels in addition to traditional risk factors (such as hypertension and dyslipidemia) may contribute to more precise risk stratification. However, it must be cautiously emphasized that our cross-sectional data cannot prove that reducing glucagon levels can prevent stroke. Establishing causality and the benefits of intervention require validation through prospective cohort studies and interventional clinical trials.

This study has several limitations that need to be considered when interpreting the results. First, and most crucially, the cross-sectional study design prevents us from inferring causality. The association we observed could have two explanations: high glucagon levels increase the risk of stroke, or the stroke event itself or its treatment (such as the use of specific medications) leads to a secondary increase in glucagon levels. Second, although we made efforts to adjust for numerous confounding factors, especially correction for medications affecting glucagon, the possibility of residual confounding cannot be completely ruled out. Moreover, duration and dose of glucose-lowering therapies affecting glucagon levels should be acknowledged. Furthermore, the study subjects were inpatients from a single tertiary hospital, and the proportions of stroke subtypes and medication use among patients vary across different regions, potentially limiting the representativeness of the population and introducing selection bias. The generalizability of the research conclusions to community populations or other ethnic groups needs further verification. Finally, peripheral blood measurements may underestimate the true physiopathological signal intensity of glucagon; future research should explore portal vein concentration detection or mathematical model correction schemes.

## Conclusion

This cross-sectional study found that among hospitalized female patients with type 2 diabetes, higher fasting glucagon levels were independently associated with the risk of comorbid stroke. Although this association was not observed in male patients, this finding suggests a potential link between glucagon and cerebrovascular diseases in women with T2DM. Future prospective studies are needed to determine the direction of this association and its underlying mechanisms.

## Supplementary materials



## Declaration of interest

The authors declare that there is no conflict of interest that could be perceived as prejudicing the impartiality of the work reported.

## Funding

This work was supported by the Tianjin Major Science and Technology Projects (grant number 21ZXJBSY00060), Tianjin Key Medical Discipline (Specialty)Construction Project (TJYXZDXK-3-002C), and Tianjin Medical University Clinical Special Disease Research Center - NeuroendocrineTumor Clinical Special Disease Research Center.

## Author contribution statement

LK, LC, and JN screened the literature and extracted data. LK, LC, JN, YL, YG, XW, SY, and WH analyzed the data. QH provided methodological guidance. LK wrote the original draft of the manuscript. QH and ML supervised the work.

## Data availability

Some or all datasets generated and/or analyzed during the current study are not publicly available but are available from the corresponding author on reasonable request.

## Ethics

This study was approved by the Ethics Institutional Review Board of Tianjin Medical University General Hospital (IRB2020-YX-027-01). Consent was obtained from each patient or subject after full explanation of the purpose and nature of all procedures used.
