# Supplementary material for: Association between glucagon and stroke in patients with type 2 diabetes
Source: Endocr Connect. 2026 Feb 17;15(2):e250791. doi: 10.1530/EC-25-0791 (PMC12920052; doi:10.1530/EC-25-0791)
Supplement: Supplementary file 1 [file supplementary_materials.pdf]

Supplementary Table 1 Clinical characteristics of patients with T2DM and T2DM&Stroke

	T2DM	T2DM&Stroke	P
Number，n（%）	1440	305	
Age，year	58 (45, 67)	66 (61, 72)	**P<0.001**
T2DM duration，month	84 (12, 180)	132 (60, 240)	**P<0.001**
Smoking，n（%）	461 (32.0)	89 (29.2)	0.322
Alcohol consumption，n（%）	387 (26.9)	77 (25.2)	0.545
Hypertension，n（%）	799 (55.5)	244 (80.0)	**P<0.001**
Fatty liver，n（%）	1023 (71.0)	192 (63.0)	**0.002**
Dyslipidemia，n（%）	1118 (77.6)	253 (83.0)	0.087
Hyperuricemia，n（%）	377 (26.2)	75 (24.6)	0.476
Lipid-lowering drug，n（%）	467 (43.4)	165 (54.1)	**P<0.001**
Antihypertensive agents，n（%）	619 (43.0)	201 (65.9)	**P<0.001**
DPP-4i，n（%）	331 (23.0)	71 (23.3)	0.912
GLP-1RA，n（%）	181 (12.6)	39 (12.8)	0.921
Insulin，n（%）	722 (50.1)	174 (57.0)	**0.028**
BMI，Kg/m2	26.23 (23.53, 29.21)	25.30 (23.42, 27.52)	**P<0.001**
BP，mmHg			
SBP	130 (121, 144)	136 (125, 147)	**P<0.001**
DBP	81 (75, 89)	80 (75, 89)	0.577
TC，mmol/L	4.84 (4.06, 5.68)	4.31 (3.45, 5.02)	**P<0.001**
TG，mmol/L	1.80 (1.29, 2.76)	1.64 (1.13, 2.22)	**P<0.001**

HDL-C，mmol/L	0.97 (0.83, 1.12)	0.94 (0.81, 1.12)	0.326
LDL-C，mmol/L	2.71 (2.13, 3.33)	2.32 (1.76, 2.92)	**P<0.001**
SUA，umol/L	334 (273, 408)	328 (262, 395)	0.193
eGFR, mL/min/1.73 m^2	103.74 (94.73, 113.70)	95.23 (84.40, 102.57)	**P<0.001**
HbA1c，%	8.2 (7.0, 9.9)	7.9 (7.03, 9.1)	0.052

Data are presented as mean ± SD, median (Q1, Q4), or number (%).

T2DM: type 2 diabetes mellitus; T2DM&Stroke: type 2 diabetes mellitus complicated by Stroke; DPP-4i: dipeptidyl peptidase 4 inhibitors;GLP-1RA: Glucagon Like Peptide-1 receptor agonists; BMI: body mass index; BP: blood pressure; SBP: systolic blood pressure; DBP: diastolic blood pressure; TC: total cholesterol; TG: triglyceride; HDL-C: high density lipoprotein cholesterol; LDL-C: low density lipoprotein cholesterol; SUA: serum uric acid; HbAlc: glycosylated hemoglobin.

Supplementary Table 2 Cases of patients in different Quartiles of glucagon levels

	Quartiles of glucagon levels			
	**Q1**	**Q2**	**Q3**	**Q4**
**Female**				
T2DM	150	148	139	130
T2DM&Srtoke	21	23	32	40
**Male**				
T2DM	214	212	219	228
T2DM&Srtoke	52	53	44	40

Supplementary Table 3 VIF of patients with T2DM and T2DM&Srtoke

	VIF	
	Female	Male
Age	2.06	1.72
T2DM duration	1.04	1.03
Smoking	1.10	1.22
Alcohol consumption	1.09	1.22
Hypertension	2.23	1.86
Fatty liver	1.29	1.18
Dyslipidemia	1.09	1.08
Antihypertensive agents	2.17	1.92
DPP-4i	1.07	1.06
GLP-1RA	1.09	1.10
Insulin	1.46	1.44
BMI	1.39	1.08
eGFR	1.86	1.64
HbA1c	1.38	1.35
FINS	1.19	1.15

DPP-4i: dipeptidyl peptidase 4 inhibitors;GLP-1RA: Glucagon Like Peptide-1 receptor agonists; BMI: body mass index; HbAlc: glycosylated hemoglobin; FINS: fasting insulin.
